# Efficacy and Safety of Amivantamab in Advanced or Metastatic EGFR-Mutant Non-Small Cell Lung Cancer: A Systematic Review

**DOI:** 10.3390/jcm13185489

**Published:** 2024-09-16

**Authors:** Ionas Papassotiriou, Antonios Kapogiannatos, Christos Makatsoris, Sabrina Bakogeorgou, Ioanna Mantogiannakou, Emmanouela Roussou, Georgios Souras, Dimitris Liakas, Theodoros N. Sergentanis, Maria Gavriatopoulou, Ioannis Ntanasis-Stathopoulos

**Affiliations:** 1 Department of Clinical Therapeutics, School of Medicine, National and Kapodistrian University of Athens, 11528 Athens, Greece; iwn.papas@gmail.com (I.P.);; 2 Department of Public Health Policy, University of West Attica, 11521 Athens, Greece

**Keywords:** lung cancer, non-small cell lung cancer, NSCLC, amivantamab, EGFR, efficacy

## Abstract

**Objectives:** This systematic review aimed to examine the efficacy and safety profile of amivantamab in patients with advanced or metastatic non-small cell lung cancer (NSCLC) and EGFR mutations. **Methods:** Three scientific databases, PubMed, Cochrane library and ClinicalTrials.gov were searched for relevant articles up until 30 June 2024. Progression-free survival (PFS), overall survival (OS), objective response rate (ORR) and ≥3 grade adverse events (AE) were the outcomes of interest. **Results:** Five clinical trials were included in this systematic review, reporting data from 1124 patients (safety population; n = 1091 efficacy population), who received amivantamab as a monotherapy or in combination with other treatments, both in a first-line and in a relapsed/refractory setting. The median PFS for groups of patients that received amivantamab ranged from 4.3 to 8.3 months, while the lowest observed OS was 10.2 months. The ORR ranged from 30% to 73%. The rate of grade 3 or higher AEs ranged from 35% to 92%, while serious AEs ranged from 29% to 52%. Infusion-related reactions (IRRs) ranged from 42% to 78% among patients that received amivantamab intravenously, while a 13% IRR rate was found in a group of patients that received amivantamab subcutaneously. **Conclusions:** Current evidence suggests that amivantamab is an effective treatment option for patients with advanced or metastatic NSCLC with EGFR mutations. Amivantamab-based combinations may prolong survival both in the treatment of naïve patients and those who have progressed on chemotherapy or tyrosine kinase inhibitors.

## 1. Introduction

Lung cancer is among the most common types of cancer worldwide, in both males and females, with 2 million new cases each year [1]. Its high mortality has been declining in the last decades as a result of advances in drug discovery and a better understanding of its pathogenesis, which has led to targeted therapies; however, it still remains a common cause of cancer death [2]. Non-small cell lung cancer (NSCLC) is the most common type of lung cancer, accounting for about of 85% of all lung cancer cases [3]. Management of NSCLC in each step of disease progression remains a major issue since its prognosis remains poor for most patients; about 60% of NSCLC patients have either advanced or metastatic cancer at diagnosis [4]. This can be partially attributed to late diagnosis, which can be resolved with pre-symptomatic screening and public awareness, but the lack of highly effective treatments against NSCLC is currently the main problem [5]. Surgical therapy with or without chemotherapy can be effective in the early stages (I and II) of NSCLC [3,5]. However, in practice, many patients are diagnosed in its late stages, when the anticipated benefit from chemotherapy and radiotherapy is modest, with 5-year survival at stage IV being approximately 6% [5].

Recent advances in understanding the pathophysiology of NSCLC subtypes and the introduction of tyrosine kinase inhibitors (TKIs) have improved survival rates in patients with NSCLC [6]. In addition, the evolution of current knowledge in this type of cancer has led us closer to precision medicine. Molecular diagnostics are currently used to identify specific mutations and translocations in patients with NSCLC to provide more effective and personalized therapies [7]. For example, EGFR mutations, which are present in 10–20% of Caucasian and >50% in Asian NSCLC patients [8,9], play a key role in both the development and treatment response in NSCLC and define the treatment plan for these patients [6]. EGFR is a tyrosine kinase receptor with a crucial role in cell proliferation and survival. Mutations in the EGFR gene are crucial in NSCLC pathology, since they provoke uncontrolled growth of cells and, therefore, contribute to the development of NSCLC.

The approval of TKIs in NSCLC has revolutionized the treatment landscape both at diagnosis and in relapse. Erlotinib, dacomitinib, gefitinib and other first or second generation TKIs are eligible for use in these patients, while osimertinib, a third generation TKI, is usually considered as a first-line therapy [10]. Furthermore, amivantamab, a human bispecific antibody that targets EGFR and mesenchymal–epithelial transition factor (MET), has gained a lot of attention due to initially promising results [11]. This molecule gained its first approval from the Food and Drug Administration (FDA) on May 2021 based on results from the CHRYSALIS study, a non-randomized and open-label multicenter clinical trial [12]. At that point, amivantamab was approved for locally advanced or metastatic NSCLC in patients with EGFR exon 20 insertion mutations who progressed on or after platinum-based chemotherapy [11]. Three years after its first approval, in March 2024, amivantamab was granted another approval from the FDA as a first-line treatment combined with carboplatin and pemetrexed for the same indication. This second approval was based on the PAPILLON study, a randomized, open-label trial with 308 NSCLC patients with EGFR exon 20 insertion mutations [13].

Despite results from the phase I CHRYSALIS trial and the phase III PAPILLON trail, there are several other ongoing trials examining the role of amivantamab in NSCLC patients with specific mutations. Recently, the results from another cohort of the CHRYSALIS trial have been published [14], as well as the results from the phase III MARIPOSA trials [15]. However, to the authors’ knowledge, there is no published systematic review or meta-analysis that combines the results of these studies. Therefore, our aim was to systematically examine the efficacy and safety of amivantamab in adult patients with advanced or metastatic EGFR-mutant NSCLC.

## 2. Materials and Methods

### 2.1. Search Strategy

This meta-analysis complies with the PRISMA (Preferred Reporting Items for Systematic Reviews and Meta-analyses) statement for systematic reviews and meta-analyses, and the protocol has been registered to the Open Science Framework “https://archive.org/details/osf-registrations-9vmp3-v1 (accessed on 27 June 2024)”.

Three scientific databases, PubMed, Cochrane library and ClinicalTrials.gov were searched for relevant articles up until 30 June 2024. The search strategy involved the use of the following main search terms in several combinations: “amivantamab”, “non-small cell lung cancer” and “NSCLC”. Language or time restrictions were not applied in the search strategy. For each article identified in the primary electronic search, the title and abstract were reviewed independently by two investigators (I.P. and A.K.). An extra search was performed on the reference lists of all articles that were considered relevant according to title and abstract. The detailed search strategy is presented in the Supplementary Materials (Supplementary Table S1).

### 2.2. Eligibility Criteria

Relevant studies were included if they met the following criteria: (a) included patients with advanced NSCLC and mutated EGFR who received amivantamab as monotherapy or in combination with other drugs, (b) were phase I–III clinical trials, (c) reported results on at least one of the following outcomes: progression-free survival (PFS), overall survival (OS), objective response rate (ORR) or incidence of grade ≥ 3 adverse events. On the contrary, studies were excluded if they were: (a) published in a language other than English; (b) non-human studies; (c) case reports, observational studies or review studies; (d) phase IV studies or real word data.

### 2.3. Data Extraction and Data Synthesis

In accordance with the PRISMA protocol, two investigators (I.P. and A.K.) independently reviewed and screened each article using the pre-specified criteria and recorded the excluded literature as well as the reasons for exclusion of each study. Any deviations between the two investigators were resolved with the mediation of a third investigator (I.N.S.), whereas all authors stated their reasons for inclusion or exclusion. After the final list of eligible literature was defined, the two investigators (I.P. and A.K.) worked independently and extracted the following data from each study: author(s), year of publication, region, trial design and phase, number of participants and their characteristics, intervention and comparator arms, EGFR mutation status and reported outcomes. For quantitative synthesis, the median PFS, median OS, percentage of ORR and ≥3 grade AEs were recorded.

### 2.4. Quality Assessment

Critical appraisal was performed with the Cochrane risk of bias tool (RoB) [16]. This tool assesses seven domains: sequence generation during randomization, allocation concealment, blinding of participants and personnel, blinding of outcome assessors, incomplete outcome data, selective reporting and other biases. Each domain was scored as low, unclear or high risk of bias, while discrepancies between authors were resolved through discussion and agreement.

## 3. Results

### 3.1. Study Characteristics

**Figure 1:**
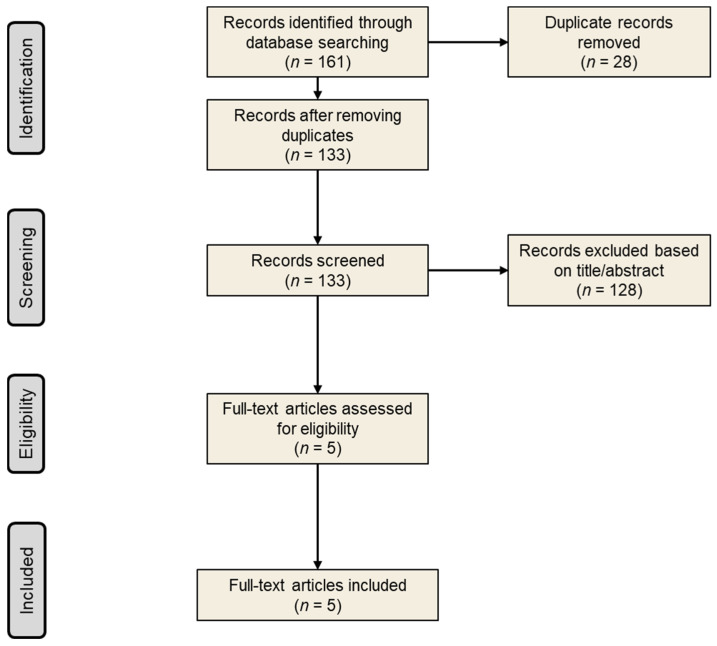
Study selection process.

From the 161 records identified through the search strategy, 5 records were reviewed in full (Figure 1). From these 5 records, all were included in the current systematic review and provided data for efficacy in 1091 patients and for safety in 1124 patients, who received amivantamab either as a monotherapy or in combination with other treatments [12,13,14,15,17]. Two studies were phase I clinical trials that reported efficacy and safety data from two different cohorts of the CHRYSALIS study [12,14]. The other three studies were phase III randomized clinical trials that reported results from three separate trials: PAPILLON, MARIPOSA-2 and PALOMA-3 [13,15,17].

The two cohorts from the phase I CHRYSALIS trial were NSCLC patients that received either amivantamab as a monotherapy after chemotherapy [12], or amivantamab in combination with 240 mg of lazertinib, taken orally, who relapsed on TKI monotherapies but were chemotherapy-naïve [14]. In both studies, amivantamab was given intravenously at a dose of 1050 mg or 1400 mg if patients were ≥80 kg.

In the PAPILLON study, a higher dose of amivantamab was given weekly as a first-line treatment, which started at 1400 mg (or 1750 if ≥80 kg) and increased to 1750 mg (or 2100 mg if ≥80 kg) after 7 weeks and was administered every 3 weeks until disease progression. This study included two arms: a group of patients that received amivantamab and chemotherapy and a group that received only chemotherapy. In both groups, chemotherapy consisted of carboplatin (5AUC) for up to four cycles and pemetrexed (500 mg/m^2^) until disease progression [13].

In the MARIPOSA-2 trial, the same dosing scheme as in the PAPILLON trial of amivantamab and chemotherapy was administered, but in patients who had progressed after or during treatment with osimertinib administered as first-line therapy or at relapse. However, this trial included three arms; one that received amivantamab in combination with 240 mg of lazertinib and chemotherapy, one that received amivantamab and chemotherapy and one that received only chemotherapy [15].

On the other hand, in the PALOMA-3 trial, there were two groups of patients who progressed on or after TKIs and chemotherapy and that received amivantamab and 240 mg of lazertinib, with the difference being that amivantamab was administered subcutaneously (SC) in one arm and intravenously in the other. In the SC group, 1600 mg (2240 mg if ≥80 kg) of amivantamab was given, whereas in the IV group, a lower dose was administered (1050 mg or 1400 mg if ≥80 kg), [17]. A more detailed presentation of the baseline characteristics of each study is presented in Table 1.

### 3.2. Efficacy

All of the selected studies reported efficacy data, where PFS and OS are presented as median months and ORRs as percentages (Table 2). Trials with comparison arms also presented the HR or RR values with a reference group. The median PFS in any group that received amivantamab, either as a monotherapy or in combination with other therapies, ranged from 4.3 (95% CI: 4.1–5.7) to 11.4 (95% CI: 9.8–13.7) months. The lowest median PFS was recorded when amivantamab was administered intravenously in combination with lazertinib in the PALOMA-3 study and is similar to the results reported from cohort E of the CHRYSALIS trial, where a combination of amivantamab and lazertinib was also administered (median PFS: 4.9 months, 95% CI: 3.7–9.5). A higher median PFS was reported in the PAPILLON study in the arm that received a combination of amivantamab and chemotherapy (median PFS: 11.4 months, 95% CI: 9.8–13.7). Results from the PAPILLON and MARIPOSA-2 study that compared amivantamab (in any combination) with chemotherapy alone revealed a similarly lower risk (HR) for progression or death from any cause ranging from 0.40 (95% CI: 0.3–0.53) to 0.48 (95% CI: 0.36–0.64).

Regarding OS, the median survival with amivantamab-based regimens ranged from 12.9 (95% CI: 12.9–NE) to 22.8 (95% CI: 14.6–NE) months. Interestingly, the PALOMA study showed that subcutaneous administration of amivantamab led to significantly prolonged OS compared to the intravenous formulation (HR = 0.62, 95% CI: 0.42–0.92). However, the median OS of the group that received the drug intravenously, as well as the median OS of the combination of amivantamab with chemotherapy in the other trials, were not reached during the reported follow-up period. So far, no statistically significant OS benefit has become evident from the addition of amivantamab to chemotherapy over chemotherapy alone (Table 2).

Overall, the ORR ranged from 30% (95% CI: 24–47) to 73% (95% CI: 65–80) in the PAPILLON study, where amivantamab was combined with chemotherapy. It was also found that patient groups that received amivantamab with chemotherapy had significantly higher ORRs in comparison to chemotherapy alone, with RRs (ORs) ranging from 1.5 (95% CI: 1.32–1.69) to 3.10 (95% CI: 2.0–4.8). The PALOMA trial also showed that subcutaneous administration was non-inferior to intravenous administration of amivantamab in terms of the ORR (RR 0.92, 95% CI: 0.70–1.23).

### 3.3. Safety

All studies provided data about adverse events (AEs), including ≥ grade 3 AEs, serious AEs (SAEs), infusion-related reactions and amivantamab-specific AEs (Table 3). The lowest percentage of at least grade 3 AEs was reported in CHRYSALIS cohort D (35%), where amivantamab was administered as a monotherapy, whereas the highest percentage (92%) was observed in a group in the PAPILLON trial that received a combination of amivantamab, lazertinib and chemotherapy. In the three groups that received a combination of amivantamab and lazertinib, the rates of ≥grade 3 AEs were similar, ranging from 50 to 56%, while the two groups that received amivantamab and chemotherapy had a frequency of ≥grade 3 AEs of more than 70%.

Among the groups that received amivantamab-based regimens, the lowest frequency of SAEs (29%) was reported in the PALOMA arm, where amivantamab was administered subcutaneously, while the highest was reported for the group that received amivantamab, lazertinib and chemotherapy. However, in six out of the seven groups that received amivantamab, the percentage of SAEs was lower than 40%.

IRRs were common among patients that received amivantamab, with a frequency ranging from 13 to 78%. The lowest frequency of IRRs (13%) was found in the subcutaneous administration of amivantamab, while the highest frequency (78%) reported in the CHRYSALIS group that received amivantamab and lazertinib. Other common amivantamab-related AEs included paronychia and rash. Paronychia was found at a frequency ranging from 37 to 56% among groups that received amivantamab, while the frequency of rashes ranged from 43 to 86%. Interestingly, a lower rate of venous thromboembolism was reported with subcutaneous (9%) compared to intravenous (14%) administration of amivantamab in the PALOMA study, where 80% of the enrolled patients in both study arms received prophylactic anticoagulation.

### 3.4. Quality Assessment

**Figure 2:**
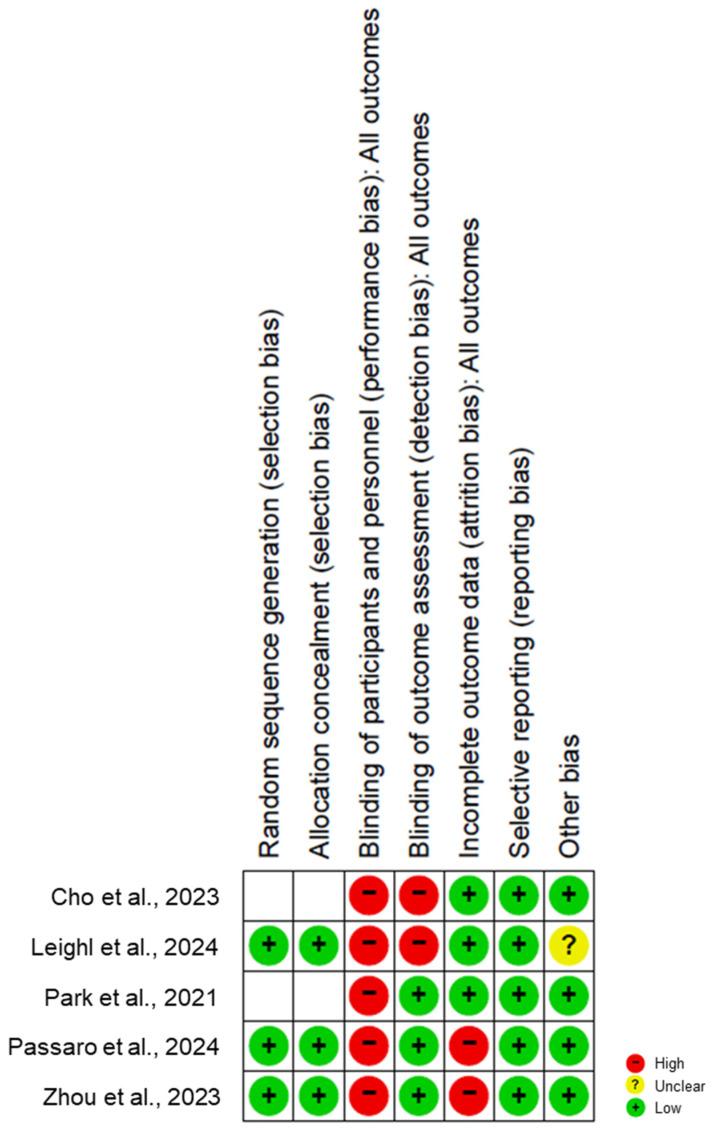
Risk of bias assessment for included studies [12,13,14,16,17].

Risk of bias assessment is presented in Figure 2, where a modified risk of bias was performed for the two phase I trials included in this review. All studies were open-label and, therefore, blinding of participants or investigators was not possible. However, most studies included assessments from a blinded independent central review as a means to minimize detection bias.

## 4. Discussion

In light of the recent FDA approval of amivantamab and the lack of a systematic review of the relevant literature, this systematic review aimed to examine the efficacy and safety of amivantamab in NSCLC patients with EGFR mutations. Available results came from five clinical trials, of which only three are randomized phase III trials. However, the dose expansion cohort of two phase I trials were included, since the first FDA approval of amivantamab was based on the CHRYSALIS trial’s primary results from its phase I study [12]. The diversity and heterogeneity of included studies did not allow for quantitative synthesis. For example, different treatments and combinations of treatments were given among the studies, while the number and type of prior treatments was also inconsistent. Study phases and designs were also heterogeneous: a seamless phase design was adopted in some of them, and despite the possible benefits of this adaptive design [18], direct comparison may prove difficult. However, despite the heterogeneity, several patterns in the efficacy and safety of amivantamab were observed and are summarized here to provide an initial assessment of this newly introduced drug in the therapeutic landscape of NSCLC.

PFS is a key outcome in cancer studies and was set as the primary outcome in this systematic review. From the available data, it was observed that amivantamab, either as a monotherapy [12] or in combination with chemotherapy [13,15] led to a high median PFS, ranging from 6.3 to 11.4 months. On the other hand, when amivantamab was only combined with lazertinib [14,17], a lower relative PFS was observed (from 4.3 to 6.1). The two groups of patients that received amivantamab intravenously in combination with lazertinib had a median PFS of less than 5 months, while the higher PFS (6.1 months) was observed in a group of patients that received amivantamab subcutaneously. However, we cannot conclude that there is any effect in the route of administration, since this was examined only in a single non-inferiority study and the difference was found to be non-significant [17]. On the other hand, it should be noted that, in the MARIPOSA trial, the group that received amivantamab, lazertinib and chemotherapy had the best PFS outcome (8.3 months) in comparison to amivantamab plus chemotherapy or chemotherapy alone. Importantly, it seems that the addition of amivantamab to chemotherapy after prior exposure to osimertinib significantly enhances PFS outcomes for this challenging patient population [15]. In addition, all three groups of patients who received amivantamab with chemotherapy (with or without lazertinib), and whose outcomes were compared with chemotherapy alone, were found to have a significantly lower hazard of disease progression or death, ranging from 0.40 to 0.48, regardless of prior lines of treatment [13,15].

Conclusions about overall survival are not easy to make since the follow-up period of most studies was not long enough and the survival data were not mature enough. The lowest median OS observed was 12.9 months and was reported in the group of patients that received amivantamab subcutaneously in combination with lazertinib [17], resulting in a much lower OS than the median OS reported in the chemotherapy only group in the PAPILLON study [13]. However, in a later study, patients received amivantamab as a first-line therapy while, in the PALOMA study, patients were receiving 2nd+ lines of therapy. Three groups of patients, one from the PAPILLON and two from the MARIPOSA trial, who received amivantamab-based combinations compared to chemotherapy alone, were found to have non-significant differences in terms of OS [13,15]. While patients in the PAPILLON study had not previously received systemic therapy, the MARIPOSA trial’s patients had already received osimertinib as a 1st or 2nd+ line of therapy.

A great heterogeneity in ORRs was found among groups of patients that received amivantamab (alone or in any combination), ranging from 30 to 73%, while ORRs followed a similar pattern to PFS. This means that, when amivantamab was combined with lazertinib without chemotherapy, a lower ORR were observed, ranging from 30–36%, whereas when amivantamab was combined with chemotherapy, higher ORRs were recorded, ranging from 63 to 73%. In addition, the positive effect of amivantamab plus chemotherapy on ORRs was found in one study where patients had not received prior systemic therapies [13] and in another study where patients had received multiple prior therapies, including TKIs [15]. This may indicate that the beneficial effect of combining amivantamab with chemotherapy compared to chemotherapy alone is evident regardless of prior lines of treatment in patients with EGFR-mutant NSCLC.

Regarding safety, the combination of amivantamab with lazertinib led to lower frequencies of ≥3 grade AEs, ranging from 52 to 56%, in comparison to a combination of amivantamab with chemotherapy, ranging from 72 to 75%. As expected, the higher frequency of ≥3 grade AEs (92%) reported from the group of patients in the MARIPOSA trial that received a combination of amivantamab, lazertinib and chemotherapy [15]. Serious AEs were also frequent, ranging from 29 to 52%, whereas a higher frequency (52%) was observed when amivantamab was combined with both lazertinib and chemotherapy. On the other hand, IRRs, which is are AEs related with the route of administration, were observed in more than 50% of patients in most studies. Patients that received amivantamab in combination with lazertinib had a slightly higher frequency of IRRs (from 66 to 78%), in comparison to those that received amivantamab and chemotherapy (from 42 to 58%). Unexpectedly, the group that received amivantamab with both lazertinib and chemotherapy had lower IRRs (56%) than groups that received the combination without chemotherapy. A very low IRR rate (13%) was achieved by subcutaneous administration of amivantamab. Therefore, given the non-inferiority of this method of administration compared to intravenous administration, subcutaneous administration could be considered a possible alternative, both in future studies and in clinical practice.

Furthermore, we included two AEs with special interest for patients receiving amivantamab treatment: paronychia and rash [19]. The frequency of paronychia was similar for most studies and groups that received amivantamab, ranging from 37 to 56%, while the combination of amivantamab with either chemotherapy or lazertinib did not clearly differentiate the occurrence of paronychia. On the other hand, a higher percentage of patients that received amivantamab developed rashes, ranging from 43 to 86%. However, the higher percentages (80% and 86%) were reported in the two CHRYSALIS cohorts [12,14], while the rest of the studies reported a frequency of rashes ranging from 43 to 54%. Last, but not least, the low rates of venous thromboembolism in the PALOMA trial underlines the importance of personalized risk evaluation as a baseline and prompt administration of anticoagulants in patients with EGFR-mutant NSCLC.

The flat dosage of amivantamab in the included studies was 1050 mg in the CHRYSALIS and PALOMA trials (1600 mg in the SC arm), while the PAPILLON and MARIPOSA trials used 1400 mg. However, no clear pattern in the dose of amivantamab and its efficacy or safety outcomes was observed.

In addition to anti-EGFR TKIs and amivantamab, anti-EGFR monoclonal antibodies (mAb) have been approved for EGFR-driven cancers [20]. Anti-EGFR mAbs have been found to be effective, especially in combination with other mAbs or TKIs, in TKI-resistant cancers and advanced NSCLC [21]. Although there are no currently published data providing a direct comparison between amivantamab and other anti-EGFR mAbs on advanced or metastatic NSCLC, the efficacy seems to be comparable [22]. However, accumulating evidence supports amivantamab both in the upfront and the relapsed/refractory setting, with remarkable clinical activity that may lead to regulatory approvals and amivantamab-based combinations being integrated into clinical practice.

During the disease course, patients on TKIs will eventually acquire resistance to these drugs, and the mechanisms by which amivantamab may overcome resistance are possibly related to its ability to overcome these resistance mechanisms [23]. Although the mechanisms of TKI resistance in NSCLC patients is not fully explored, there is evidence that receptor tyrosine kinases (RTKs), like MET and the MERTK ligand GAS6, may play a role [24,25]. Amivantamab was chosen among several anti-EGFR molecules since it also acts as an anti-MET factor [26]. Its binding affinity to both EGFR and MET is adequate to reduce their expression and overcome drug resistance acquired due to TKIs [23]. Therefore, amivantamab’s main mechanism of action lies in its ligand blocking ability, which prevents EGFR and MET receptors from binding with their ligands and, thus, inhibits signal pathways [26,27]. In addition, the immunoregulatory activity of amivantamab has also been found in preclinical studies, showing that it can activate and direct immune cells to attack tumor cells [28].

Therefore, as it happens with TKIs and mAbs, patients on amivantamab will also acquire drug resistance, which is a problem with all targeted therapies in cancer. Although the exact pathways are not the same across different types of cancer, targeted therapies for lung cancer have been found to induce expression of cytosine deaminases like APOBECs (apolipoprotein B mRNA editing enzymes, catalytic polypeptide-like) [29,30]. APOBECs have an important role in genomic and epigenomic editing [31], which in turn alter the response to targeted therapies and drive the development of drug-tolerant persister (DTP) cells [30]. These cells, which constitute a subpopulation of tumor cells, are responsible for drug resistance in targeted therapies. DTP cells develop several survival mechanisms against therapy, while their high phenotypic heterogeneity is indicative of their adaptability [32]. It has been shown that the evolution of DTP cells is quite common in lung cancer and is highly attributed to APOBEC’s effect [30]. In addition to this mechanism, Anexelekto (AXL) has been found to mediate drug resistance in many cancers, while it is also involved in TKI resistance in EGFR-mutant NSCLC, probably by sharing the same signaling pathway with EGFR [33]. AXL also induces low fidelity polymerases, which have been identified as molecules that possibly play a role in acquired drug resistance [34]. These enzymes copy DNA with low accuracy, which makes them prone to errors during replication, leading to higher mutation rates [35]. These conditions facilitate tumor cell adaptations to pressure from targeted therapies, which finally enable drug resistance [34,36].

Another group of RTKs, which have been shown to play a role in EGFR TKI resistance, is the human epidermal growth factor receptor (HER) family, consisting of four members [37]. HER3 not only displays persistent signaling, but can also form heterodimers similar to EGFR and, through activation of several downstream signaling pathways, favors tumor cell survival and drug resistance [38]. However, available data of the interaction and/or effect of amivantamab on these drug resistance mechanisms are scarce. A better understanding of interactions between targeted therapies and drug resistance mechanisms in lung cancer is essential and will lead to a new era of combination therapies that would also target key drug resistance molecules [39].

This systematic review provided evidence from five clinical trials, where a benefit of amivantamab on PFS is clear in all studies. Amivantamab-based regimens have a role both in the upfront setting and, especially, in the relapsed/refractory setting after prior TKI failure in patients with EGFR-mutant NSCLC [40,41]. The potential OS benefit has not been demonstrated yet, possibly due to the short follow-up. However, the limited data and the differences in drug combinations and dosages of amivantamab between available clinical studies are important limitations to drawing further conclusions. Moreover, the included studies were very heterogenous in key study characteristics, such as study design, treatments, comparison groups, and the number and type of prior therapies. Evidence from the available studies does not allow for a direct comparison of efficacy or safety of amivantamab with other modern drugs against relapsed EGFR-mutated NSCLC.

More than 20 clinical trials are currently registered in ClinicalTrials.gov that examine the role of amivantamab against NSCLC in several combinations and comparisons. Therefore, more evidence will become available in the future, and we hope that more robust conclusions will be made in order to incorporate this new drug into everyday clinical practice for patients with EGFR-mutant NSCLC.

## Figures and Tables

**Table 1:** Baseline characteristics of the studies included in the systematic review.

													EGFR Mutation		
First Author/Year	Region	Study Design/Phase	Setting	Arms/Groups	Sample Size (n)	Age, Median (Range)	Ethnicity	Females n (%)	Smokers n (%)	NSCLC Subtype n (%)	Performance Status (ECOG) n (%)	History of Brain Metastasis n (%)	Exon 19 Deletion n (%)	Leu858Arg n (%)	Interventions
[12]	South Korea, Japan, United States	CHRYSALIS/Phase I (dose expansion cohort D), open-label	EGFR mutated patients with locally advanced or metastatic NSCLC, progressed on (or ineligible for, or declined) platinum-based chemotherapy	Dose expansion (efficacy) group	81	Efficacy population: 62 (42–84)	Multiple	Efficacy population: 48 (59)	38 (47)	Adenocarcinoma: 77 (95) Squamous cell carcinoma: 3 (4) Others: 1 (1)	0: 26 (32) 1: 54 (67) 2: 1 (1)	18 (22)	NG	NG	1050 mg Ami (or 1400 mg if ≥80 kg)
				Safety group	114	NG	NG	NG	NG	NG	NG	NG			
[14]	International	CHRYSALIS/Phase I (dose expansion cohort E), open-label	EGFR mutated patients with locally advanced or metastatic NSCLC, progressed on osimertinib as 1st or 2nd line treatment	Dose expansion group	45	65 (39–85)	Multiple	25 (56)	20 (44)	NG	0: 12 (27) 1: 33 (73)	13 (29)	30 (67)	14 (31)	1050 mg Ami (or 1400 mg if ≥80 kg) + 240 mg laz
[13]	International	PAPILLON/Phase 3, open-label	No previously treated EGFR mutated patients with locally advanced or metastatic NSCLC	Ami + Chemo	153	61 (27–86)	Multiple	85 (56)	65 (42)	Adenocarcinoma: 151 (99) Large cell carcinoma: 0 (0) Others: 2 (1)	0: 54 (35) 1: 99 (65)	35 (23)	NG	NG	1400 mg Ami (or 1750 if ≥80 kg) and 1750 mg (or 2100 mg if ≥80 kg) after 7 weeks + Chemo
				Chemo	155	62 (30–92)	Multiple	93 (60)	64 (41)	Adenocarcinoma: 153 (99) Large cell carcinoma: 1 (1) Others: 1 (1)	0: 55 (35) 1: 100 (65)	36 (23)	NG	NG	Carboplatin (5 mg/mL/min) + pemetrexed (500 mg/m^2^ of BSA)
[17]	International	MARIPOSA, Phase 3, open-label	EGFR mutated patients with locally advanced or metastatic NSCLC, progressed on or after oscimertinib	Ami + Laz + Chemo	263	61 (23–83)	Multiple	168 (64)	87 (33)	Adenocarcinoma: 260 (99) Other: 3 (1)	0: 92 (35) 1: 171 (65)	120 (46)	165 (63)	98 (37)	1400 mg Ami (or 1750 if ≥80 kg) and 1750 mg (or 2100 mg if ≥80 kg) after 7 weeks + 240 mg Laz + Chem
				Ami + Chemo	131	62 (36–84)	Multiple	81 (62)	41 (31)	Adenocarcinoma: 130 (99) Other: 1 (1)	0: 55 (42) 1: 76 (58)	58 (44)	89 (68)	42 (32)	Ami + Chemo
				Chemo	263	62 (31–85)	Multiple	157 (60)	95 (36)	Adenocarcinoma: 260 (99) Other: 3 (1)	0: 101 (38) 1: 162 (62)	120 (46)	183 (70)	79 (30)	Carboplatin (5 mg/mL/min) + pemetrexed (500 mg/m^2^ of BSA)
[16]	International	PALOMA/Phase 3, open-label	EGFR mutated patients with locally advanced or metastatic NSCLC, progressed on or after oscimertinib (or other 3rd generation TKIs) and platinum-based therapy	Subcutaneous (SC)	206	61 (35–82)	Multiple	138 (67)	65 (32)	Adenocarcinoma: 204 (99) Large cell carcinoma: 1 (0.5) Squamous cell carcinoma: 1 (0.5) Other: 0 (0)	0: 58 (28) 1: 148 (72)	70 (34)	135 (66)	71 (34)	1600 mg Ami (or 2240 mg if≥80 kg weight) + 240 mg Laz
				Intravenous (IV)	212	62 (29–81)	Multiple	141 (67)	67 (32)	Adenocarcinoma: 207 (98) Large cell carcinoma: 1 (0.5) Squamous cell carcinoma: 3 (1), Other: 1 (0.5)	0: 61 (29) 1: 151 (71)	72 (34)	138 (65)	74 (35)	1050 mg Ami (or 1400 mg if ≥8 kg weight) + 240 mg Laz

Ami: amivantamab, Laz: lazertinib, Chemo: chemotherapy, ECOG: Eastern Cooperative Oncology Group performance status, EGFR: epidermal growth factor receptor, NSCLC: non–small cell lung cancer, BSA: body surface area.

**Table 2:** Efficacy outcomes of the selected trials.

Study	Arms	No of Patients	PFS		OS		ORR	
			Median (95% CI)	HR (95% CI); *p*	Median (95% CI)	HR (95% CI); *p*	% (95 CI)	RR (96% CI)
[12]	Single (Ami)	81	8.3 (6.5–10.9)	-	22.8 (14.6–NE)	-	40 (29–51)	-
[14]	Single (Ami + Laz)	45	4.9 (3.7–9.5)	-	NE	-	36 (22–51)	-
[13]	Ami + Chemo	153	11.4 (9.8–13.7)	0.40 (0.3–0.53); *p* < 0.001	NE	0.67 (0.42–1.09); *p* = 0.11	73 (65–80)	1.50 (1.32–1.68); *p* < 0.001
	Chemo	155	6.7 (5.6 7.3)	Ref	24.4 (22.1–NE)	Ref	47 (39–56)	Ref
[17]	Ami+ Laz+ Chemo	263	8.3 (6.8–9.1)	0.44 (0.35–0.56); *p* < 0.001	NG	0.96 (0.67–1.35); *p* = 0.80	63 (57–69)	2.97 * (2.08–4.24); *p* < 0.001
	Ami + Chemo	131	6.3 (5.6–8.4)	0.48 (0.36–0.64); *p* < 0.001	NG	0.77 (0.49–1.21); *p* = 0.25	64 (55–72)	3.10 * (2.00–4.80); *p* < 0.001
	Chemo	263	4.2 (4.0–4.4)	Ref	NG	Ref	36 (30–42)	Ref
[16]	SC (Ami + Laz)	206	6.1 (4.3–8.1)	0.84 (0.64–1.10); *p* = 0.20	12.9 (12.9–NE)	0.62 (0.42–0.92); *p* = 0.02	30 (24–37)	0.92 (0.70–1.23)
	IV (Ami + Laz)	212	4.3 (4.1–5.7)	Ref	NE (10.2–NE)	Ref	33 (26–39)	Ref

PFS: progression-free survival, OS: overall survival, ORR: objective response rate, HR: hazard ratio, RR: relative risk, NE: not estimable, NG: not given, Ref.: reference. * Values presented as odds ratios (ORs).

**Table 3:** Safety outcomes of the selected trials.

Study	Arm	Adverse Events n (%)				
		3 ≥ grade	Serious	IRR	Paronychia	Rash
[12]	Single (Ami)	40 (35)	34 (30)	75 (66)	51 (45)	98 (86)
[14]	Single (Ami + Laz)	25 (56)	17 (38)	35 (78)	22 (49)	36 (80)
[13]	Ami + Chemo	114 (75)	56 (37)	63 (42)	85 (56)	81 (54)
	Chemo	83 (54)	48 (31)	2 (1)	0 (0)	12 (8)
[17]	Ami + Laz + Chemo	242 (92)	137 (52)	148 (56)	133 (51)	126 (48)
	Ami + Chemo	94 (72)	42 (32)	76 (58)	48 (37)	56 (43)
	Chemo	117 (48)	49 (20)	1 (0.4)	1 (0.4)	12 (5)
[16]	SC (Ami + Laz)	107 (52)	59 (29)	27 (13)	111 (54)	95 (46)
	IV (Ami + Laz)	118 (56)	64 (30)	138 (66)	108 (51)	91 (43)

Ami: amivantamab, Laz: lazertinib, Chemo: chemotherapy, SC: subcutaneous, IV: intravenous, IRR: infusion-relation reaction.

## Data Availability

Data presented in this study are available upon reasonable request.

## Supplementary Materials

The following supporting information can be downloaded at: https://www.mdpi.com/article/10.3390/jcm13185489/s1, Table S1: Research Algorithm of Each Database.
