# Supplementary material for: Efficacy and Safety of Amivantamab in Advanced or Metastatic EGFR-Mutant Non-Small Cell Lung Cancer: A Systematic Review
Source: J Clin Med. 2024 Sep 16;13(18):5489. doi: 10.3390/jcm13185489 (PMC11432208; doi:10.3390/jcm13185489)
Supplement: Supplementary file 1 [file jcm-13-05489-s001.zip › jcm-3188314-supplementary.pdf]

Supplementary Material

Table S1

Research algorithm of each database

Database	Algorithm
PubMed	"amivantamab"[Title/Abstract] AND ("non small cell lung cancer"[Title/Abstract] OR "NSCLC"[Title/Abstract])
Cochrane	Amivantamab AND (non small cell lung cancer) OR ("NSCLC)
Clinicaltrials.gov	(Non-small Cell Lung Cancer OR NSCLC) AND Amivantamab
